# Symmetry and spatial distribution of muscle glucose uptake in the lower limbs during walking measured using FDG-PET

**DOI:** 10.1371/journal.pone.0215276

**Published:** 2019-04-29

**Authors:** Sjoerd Kolk, Edzo Klawer, Eric Visser, Daphne Lobeek, Jan Schepers, Nico Verdonschot, Vivian Weerdesteyn

**Affiliations:** 1 Radboud University Medical Center, Donders Centre for Neuroscience, Department of Rehabilitation, Nijmegen, The Netherlands; 2 Radboud University Medical Center, Radboud Institute for Molecular Life Sciences, Department of Radiology and Nuclear Medicine, Nijmegen, The Netherlands; 3 Materialise N.V., Leuven, Belgium; 4 Radboud University Medical Center, Radboud Institute for Health Sciences, Orthopaedic Research Laboratory, Nijmegen, The Netherlands; 5 Laboratory for Biomechanical Engineering, University of Twente, Enschede, The Netherlands; 6 Sint Maartenskliniek Research, Nijmegen, The Netherlands; Universidade Estadual Paulista Julio de Mesquita Filho, BRAZIL

## Abstract

**Purpose:**

This study aimed to elucidate whether muscle activity (in terms of glucose uptake) between the legs can be considered symmetrical during walking. Furthermore, we aimed to determine whether the [^18^F]-fluorodeoxyglucose was distributed heterogeneously throughout each muscle, and if so, whether areas of high uptake would be clustered.

**Methods:**

Ten healthy participants walked on a treadmill at self-selected comfortable walking speed for a total of 90 minutes, 60 minutes before and 30 minutes after intravenous injection of 50 MBq [^18^F]-fluorodeoxyglucose. Thereafter, a positron emission tomography/computed tomography scan of the lower limb was acquired. Three-dimensional muscle contours of 78 (= 39x2) muscles of the left and right lower limb were semi-automatically determined from magnetic resonance imaging scans. After non-rigid registration, those muscle contours were used to extract [^18^F]-fluorodeoxyglucose uptake from the positron emission tomography scans.

**Results:**

Large asymmetries were observed in the lower leg muscles (e.g. median absolute asymmetry index of 42% in the gastrocnemius medialis) and in the gluteus minimus (30% asymmetry) and gluteus medius (15% asymmetry), whereas the uptake in the thighs was relatively symmetrical between the limbs (<6% asymmetry). These were not related to limb-dominance nor to inter-limb differences in muscle volume. The [^18^F]-fluorodeoxyglucose distribution was not distributed normally; most voxels had a relatively low standardized uptake value, and a minority of voxels had a relatively high standardized uptake value. The voxels with higher [^18^F]-fluorodeoxyglucose uptake were distributed heterogeneously; they were clustered in virtually all muscles.

**Conclusion:**

The findings in this study challenge the common assumption of symmetry in muscle activity between the limbs in healthy subjects. The clustering of voxels with high uptake suggests that even in this prolonged repetitive task, different spatial regions of muscles contribute differently to walking than others.

## Introduction

Walking involves a complex integration of muscular contractions with the goal of maintaining stance stability and forward progression. Walking in healthy persons has traditionally been assumed to be a symmetrical motion, either from assumption or for convenience of data collection and analysis [1]. This assumption may be considered reasonable, as most healthy persons walk without a limp or other visible evidence of asymmetry. However, several studies have found that the legs and individual muscles do not necessarily behave symmetrically during gait. For instance, asymmetries in kinematics [2, 3] and muscle activation [4, 5] have been reported. In kinematics, Maupas et al. found that 51.6% of walkers exhibited more than 5° of angular difference in sagittal knee range of motion between the left and right knees [2]. Gundersen et al. found in 12 out of their 14 subjects that at least two examined parameters showed asymmetries (for example, maximum knee extension and step length), and that the asymmetries occurred in an unpredictable fashion between the dominant and non-dominant limbs [3]. In muscle activation (using electromyography), Õunpuu et al. found that nine out of ten subjects showed significant differences between the dominant and non-dominant limb in at least three out of seven muscles tested [4]. Arsenault et al. found between-limb differences in soleus activation of up to 20% of a maximum voluntary contraction [5]. A better understanding of these asymmetries is important, for instance in determining ranges of normal (healthy) asymmetry, beyond which a patient with a certain pathology is considered to be truly asymmetrical. Also, asymmetry is an important consideration in validating musculo-skeletal models, which generally assume that healthy persons walk with equal activity levels in muscles in both legs [6, 7].

To assess muscle activation patterns, researchers typically utilize surface electromyography. This method is convenient and noninvasive, but has disadvantages such as a limited number of muscles that can be measured simultaneously, the need for a reference contraction if muscles are to be compared with each other, inability to measure deep-lying muscles and deep-lying aspects of superficial muscles, and confounding factors such as crosstalk and adipose tissue [8, 9]. In most surface electromyography applications, the electrodes are placed in discrete locations on the skin superficial to the muscle. The resulting electromyography data are generally considered to reflect the activity of the muscle as a whole. However, it is known (e.g. from multi-channel electromyography studies) that the spatial activity of muscle regions can be heterogeneous [10–15]. For example, during standing, the gastrocnemius medialis was found to be more active in the distal region than in the proximal region [14]. Moreover, the activity level of muscle regions can shift depending on exercise intensity, as was found in the gastrocnemius medialis in an isometric plantar flexion exercise at varying contraction levels [13]. Finally, it is known from positron emission tomography (PET) studies, that the overall heterogeneity of glucose uptake in muscles varies depending on the exercise intensity; the heterogeneity decreasing with increasing exercise intensity [16]. Region-specific activation may influence the magnitude and direction of forces that are applied to tendons [17], which may, for instance, have relevance in the study of injury mechanisms or muscle diseases. Insight into region-specific activation could also be useful for validating musculo-skeletal models, which often contain multiple musculo-tendon actuators per muscle, each of which with its own specific calculated activation level during a given activity (e.g. walking, stair negotiation or other activities of daily living).

Positron emission tomography with [^18^F]-fluorodeoxyglucose (FDG) is a technique that can be used to simultaneously assess glucose uptake in entire muscles during activity in both limbs, as well as region-specific differences in glucose uptake within muscles. It thus offers several advantages for assessing walking asymmetry and within-muscle activity compared to other techniques such as surface electromyography: it can measure all muscles at the same time, it can measure deep-lying muscles that would otherwise be difficult or impossible to access unless needle electrodes are inserted, and it is not hindered by crosstalk from other muscles or adipose tissue. The FDG-PET technique might, therefore, expand our understanding of asymmetries in walking and intra-muscular inhomogeneity in activation. This technique is based on the principle that contracting muscles take up glucose (and FDG) from the blood to replenish their expended energy. Although muscle cells can use various substrates (e.g. internal glycogen and triglyceride stores, plasma free fatty acids), the plasma glucose uptake is likely constant in a low intensity exercise such as walking [18]. Several authors have already used FDG-PET to investigate muscle activity during walking [19–23]. These studies found, for example, that the muscles in the lower leg were more active than those in the thigh and around the hip, and that FDG-PET could be used to assess muscle activity in deep muscles such as the gluteus minimus, iliacus, and tibialis posterior, which had previously been difficult to measure using surface electromyography [19, 20, 23]. However limited data on asymmetries between muscles in the left and right legs, and region-specific activation within muscles are available [22, 24, 25]. In part, this may have been due to the analyses of the FDG uptake data being limited to single slices of the PET scan, instead of taking the uptake in the entire muscle into account. Using three-dimensional magnetic resonance imaging (MRI) segmentation techniques, the added value of analyzing the uptake in the entire muscles was shown in previous work from our group [23].

This study’s aims were threefold. First, we aimed to elucidate whether muscle activity (in terms of glucose uptake) between the legs can be considered symmetrical during walking. Second, we aimed to determine whether the FDG uptake was heterogeneous throughout each muscle. Third, in case the uptake would be heterogeneous, we aimed to investigate whether muscle regions that exhibited high uptake would be clustered. The corresponding hypotheses were that (1) muscle activity would be largely symmetrical between the dominant and non-dominant limbs, (2) FDG uptake would be heterogeneous throughout the majority of muscles, and (3) that voxels with high uptake would be scattered throughout the muscle.

## Methods

The subjects, protocol and scans have been described in detail previously [23], and will therefore be described only briefly.

### Subjects

Ten healthy subjects with no history of major injury, orthopedic surgery on the lower limb, ailment related to carbohydrate metabolism, or cardiac or muscular disease participated in this study. There were five men and five women, age 23–60 years, height 1.60 m to 1.95 m, weight 55.5 kg to 91.7 kg. The dominant limb was determined to be the limb used to kick a ball [26, 27]. All procedures were approved by the local ethical committee and written informed consent was obtained from all participants.

### Protocol

The protocol consisted of 90 minutes of walking on a treadmill at self-selected comfortable pace; 60 minutes before and 30 minutes after injection with 53.6±1.8 MBq FDG. The walking speed was 1.26 ±0.11 m/s. The injected activity was low since the scans were made in 3D mode and through the use of time-of-flight technology on a latest generation PET scanner [28]. The exercise intensity was kept low, at a constant level, and maintained for a long time to limit the effects of varying substrate use (including plasma free fatty acids, triglyceride stores, and internal glycogen) as much as possible, with the aim of maintaining a steady state in glucose uptake from the blood. When walking ceased, subjects rested for 30 minutes, and then a positron emission tomography/computed tomography (CT) scan of the lower limb was acquired. Imaging 60 minutes after injection allowed us to have good tissue-to-background ratios with limited uptake in blood [29]. The subjects had refrained from eating and drinking anything except water for at least six hours, and from participating in strenuous physical activities for two days before the session.

### Positron emission tomography and computed tomography scans

The PET and CT scans were made with a Biograph 40 mCT (Siemens AG, Erlangen, Germany), from the feet to at least the iliac crest (2–4 bed positions). The slice thickness of the PET scan was 2.0 mm, and image reconstruction was performed with a matrix size of 512x512 and voxel size of 1.6 x 1.6 mm. The images were filtered using a 3D Gaussian filter with a kernel width of 3 mm full width at half maximum. The CT images were used both for attenuation correction (reconstruction with 5.0 mm slice thickness, B19f convolution kernel), and anatomical reference (reconstruction with 2.0 mm slice thickness, B31f convolution kernel).

### Magnetic resonance imaging scans

The MRI scans were acquired with a Magnetom Skyra (Siemens AG, Erlangen, Germany) at 3 Tesla. The slice thickness was 3.0 mm in the hip, knee and ankle regions, and 8.0 mm in the long bone regions in between, such that a higher level of detail was available in the areas where most muscles originate or insert. The scan parameters (repetition time/echo time: 450-545/9 ms) were set to achieve optimal visibility of muscle boundaries [23, 30].

### Image analysis

The end goal of the image analysis phase was to extract FDG uptake values from each of 78 muscles (39 muscles in both limbs, see [23]) from the PET scans. To achieve this, three-dimensional regions-of-interest (ROIs) that reflected the muscle boundaries were needed. Several sequential steps were taken using Mimics (Materialise N.V., Leuven, Belgium), Matlab 2012b (the Mathworks, Natick, MA, USA) and the Image Segmentation and Registration Toolkit 4.5.2 (ITK) (Kitware Inc., Clifton Park, NY, USA).

1. Using the MRI scan of the left lower limb of the first of the healthy subjects, 39 muscles were segmented manually on the axial slices in Mimics. These ‘stacked’ two-dimensional boundaries of each muscle formed three-dimensional ROIs. This complete segmentation will henceforth be referred to as ‘atlas’.
2. The muscles in the left lower limb of the other nine subjects were semi-automatically segmented in Mimics, using the atlas of muscle ROIs and an algorithm based on non-rigid registration of the MRI scans. More detail can be found in [23]. The resulting muscle segmentations were thoroughly checked and manually corrected where necessary.
3. The MRI scans and muscle ROIs of the left lower limb of all ten subjects were mirrored to their respective right lower limb, again with an algorithm based on non-rigid registration. Since the orientation of both limbs was not always perpendicular to the axial direction of the scan, and the fact that left-to-right anatomical differences might exist, the left and right limbs were treated as separate entities. Therefore, segmentation of the muscles of the right limb was checked and corrected again where necessary.
4. The MRI scan and the muscle ROIs were registered onto the CT scan, using ITK. The muscle ROIs were manually checked and corrected where necessary, this time using the CT scan as the anatomical reference. Since the CT and PET scans were taken with the subject lying on the table in the same position, no further adjustment of muscle ROIs was necessary once the muscle ROIs properly reflected the muscle contours on the CT scan.
5. The muscle ROIs were exported as DICOM (Digital Imaging and Communications in Medicine) mask files.
6. The DICOM mask files and the PET DICOM files (which were obtained directly from the PET scanner) were loaded into Matlab. Then, the ones (reflecting the muscle’s location) and zeros (all surrounding areas) in the DICOM mask files were multiplied with the PET scan (containing grey values reflecting the FDG uptake in Bq/mL). The FDG uptake values were corrected for decay time since the start of the scan using the rescale slope and intercept values extracted from the DICOM headers.

An important note is that the PET DICOM files from which we extracted the FDG uptake values were obtained directly from the Biograph 40 mCT; they were not ‘morphed’ or altered in any way.

### Analysis of asymmetry

To analyze the amount of asymmetry between the dominant and non-dominant lower limbs, we used the absolute symmetry index (ASI) and the symmetry index (SI) [31–34]. The equations for the SI and ASI of muscle *i* are:

$${SI}_{i}=\frac{{SUV}_{i,dominant}-{SUV}_{i,non-dominant}}{\frac{1}{2}({SUV}_{i,dominant}+{SUV}_{i,non-dominant})}\times 100\%,\;and\;{ASI}_{i}=|{SI}_{i}|$$

Where *SUV* is the mean Standardized Uptake Value of that muscle [35, 36]. The SUV reflects the normalized, mean FDG uptake in a muscle, and is given by the following formula for muscle *i*:

$${SUV}_{i}=\frac{Measured\;total\;activity\;in\;muscle\;(Bq)/Muscle\;volume\;(mL)}{Injected\;activity\;(Bq)/Body\;mass\;(g)}$$

We corrected the SUV for radioactive decay between the injection and the start of the scan. Since the SUV is normalized by the muscle volume, and volume differences might exist between muscles in the dominant and the non-dominant lower limb, these could have influenced the SUVs. Therefore, we also multiplied the SUV by the muscle volume, and refer to this value as the absolute uptake value (AUV).

The SI provided a measure for analyzing whether systematic differences existed in uptake between the dominant and the non-dominant limb, whereas absolute values of SI indicated the degree of non-directional asymmetries. Only the five largest muscles (in terms of volume) in each segment (pelvis, thigh, lower leg) were analyzed, since in previous work, those muscles were found to provide the largest contributions to walking [23]. Hence, in total we considered 30 muscles (15 on each side) for the analyses.

### Heterogeneous uptake

Heterogeneity in uptake within muscles was examined using the skewness of the FDG uptake. The skewness provides a measure for the symmetry of the probability distribution, which for this study was an advantage over the coefficient of variation, which only provides a measure for the spread of the distribution (the coefficients of variation have been reported previously [23]). If the distribution has equally long and thick tails to the left and to the right, the skew is zero. If the most frequently occurring FDG uptake values clustered at the lower end of the distribution and the tail pointed towards the higher scores, the skewness was positive, and vice versa for negative skew [37].

### Clustering

To examine the degree to which voxels with high SUVs were clustered in a given muscle, we first classified the voxels that were amongst those with the highest five percent uptake as being ‘hot’ voxels, and the other voxels (i.e. 95 percent of the voxels) as ‘cold’ voxels. We then calculated for each hot voxel with how many hot voxels it shared a border (number of hot neighbors–NHN). This calculation was also done for determining the number of hot voxels that bordered each cold voxel. The calculation in both cases was performed using a custom algorithm written in Matlab that detected the 6-point connectivity of the voxel. The average number of hot neighbors per hot voxel was then divided by the average number of hot neighbors per cold voxel. Since there were 19 times (= 95/5) less hot voxels than cold voxels, the denominator was multiplied by 19. This yielded a dimensionless index we will call the ‘cluster index’. The cluster index of muscle *i* is given by:

$${cluster\;index}_{i}=\frac{\frac{1}{m}\sum_{h=1}^{h=m}{NHN}_{h}}{\frac{1}{n}\sum_{c=1}^{c=n}{NHN}_{c}\times 19}$$

The numerator reflects the average number of hot voxels *m* per hot voxel *h*. The denominator reflects the average corrected number of hot voxels *n* per cold voxel *c*. A cluster index close to 0 would correspond to a situation where there is a homogeneous spread of the hot voxels throughout the muscle, whereas a higher cluster index value would indicate a tendency for hot voxels to cluster.

### Statistical analysis

The distributions of the SUV and AUV values of each of the 30 muscles were tested for normality with Kolmogorov-Smirnov tests. Since about half of these tests indicated that data were not normally distributed, we used non-parametric tests (Wilcoxon matched-pair signed-rank) for all comparisons between muscles of the dominant and the non-dominant limb. The significance level was set at *P* ≤ 0.05.

## Results

### Inter-limb asymmetries

There were large asymmetries in uptake between all muscles in both limbs, as indicated by the absolute symmetry indices (ASI) (Table 1). These differences were most pronounced in the lower leg, where median ASI values between 12% (soleus, tibialis posterior) and 42% (gastrocnemius medialis) were found. For individual subjects, differences up to 98% were found (tibialis anterior). In the hip region, the gluteus minimus and medius exhibited the largest asymmetry (median ASI of 30% and 15%, respectively). The uptake in the thighs was relatively symmetrical between the limbs; median ASI were 10% or lower in all muscles. All subjects except one had at least one asymmetry that was larger than 25% (Table 1, last column).

**Table 1:** Absolute Symmetry Indices of SUV for muscles of the lower limb during walking.

Muscle	ASI	Number of subjects >25% ASI	Subject numbers >25% ASI
Gluteus maximus	5% (0%–49%)	1	8
Gluteus medius	15% (1%–86%)	2	5, 8
Gluteus minimus	30% (1%–64%)	6	2, 5, 7, 8, 9, 10
Iliacus	8% (2%–18%)	0	-
Psoas	8% (2%–25%)	1	5
Adductor magnus	6% (1%–13%)	0	-
Vastus lateralis	8% (0%–9%)	0	-
Vastus intermedius	3% (0%–15%)	0	-
Vastus medialis	3% (0%–19%)	0	-
Rectus femoris	3% (1%–72%)	1	9
Soleus	12% (0%–65%)	3	2, 4, 9
Gastrocnemius medialis	42% (3%–93%)	7	1, 3, 4, 5, 7, 9, 10
Gastrocnemius lateralis	28% (3%–73%)	6	1, 2, 4, 8, 9, 10
Tibialis posterior	12% (3%–82%)	4	1, 2, 3, 5
Tibialis anterior	39% (3%–98%)	6	2, 3, 4, 8, 9, 10

ASI: Absolute Symmetry Index. Values are given as median (range).

**Fig 1:**
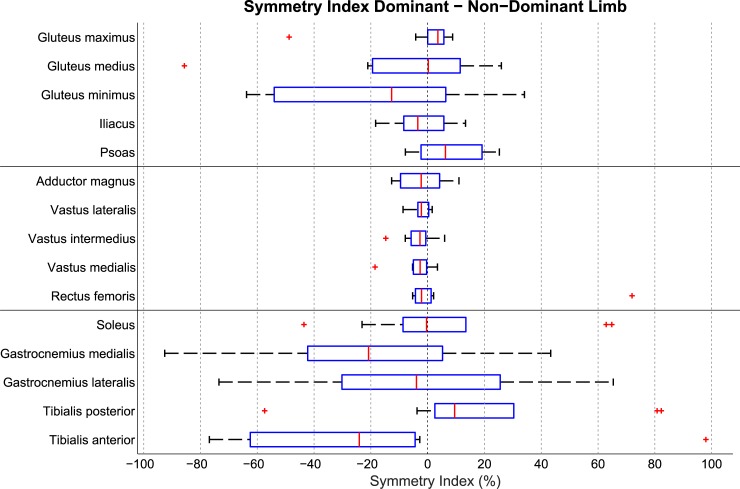
Box plot of the symmetry index. This shows the differences in uptake between the muscles in the dominant and the non-dominant lower limb. On each box, the central mark is the median, the edges of the box are the 25th (q1) and 75th (q3) percentiles, the whiskers extend to the most extreme data points not considered outliers, and outliers are plotted individually. Points are drawn as outliers if they are larger than q3 + 1.5*(q3 –q1) or smaller than q1–1.5*(q3 –q1).

With regard to the direction of these asymmetries, SUVs were not significantly different between the dominant and the non-dominant limb in 14 out of 15 muscles (Table 2). The median symmetry indices were smaller than 25% in all muscles, and smaller than 10% in 12 out of 15 muscles (Fig 1). Similar to the SUV-based comparisons, when inter-limb muscle volume differences were taken into account (AUV), none of the 15 muscles exhibited significant systematic differences in AUV between the dominant and the non-dominant limb (Table 2).

**Table 2:** Standardized and Absolute Uptake Values for muscles of the lower limb during walking.

Muscle	SUV dom. limb (g/mL)	SUV non-dom. limb (g/mL)	AUV dom. limb (g)	*P*-values SUV dom. vs. non-dom. limb*	*P*-values AUV dom. vs. non-dom. limb*
Gluteus maximus	0.62 (0.46–0.85)	0.58 (0.44–1.40)	565 (335–752)	0.20	0.65
Gluteus medius	0.85 (0.63–2.30)	0.86 (0.66–5.74)	321 (164–764)	0.88	0.33
Gluteus minimus	1.26 (0.70–5.68)	1.56 (0.82–6.67)	142 (52–798)	0.51	0.58
Iliacus	0.72 (0.57–1.00)	0.77 (0.58–1.07)	138 (95–209)	0.51	0.29
Psoas	0.76 (0.50–1.01)	0.68 (0.54–0.93)	128 (56–171)	0.09	0.09
Adductor magnus	0.72 (0.54–1.02)	0.75 (0.53–0.94)	359 (240–636)	0.80	0.96
Vastus lateralis	0.64 (0.44–0.68)	0.64 (0.46–0.73)	420 (268–592)	0.06	0.06
Vastus intermedius	0.71 (0.49–0.79)	0.75 (0.52–0.89)	311 (237–623)	**0.05**	0.24
Vastus medialis	0.66 (0.45–0.82)	0.68 (0.45–0.99)	270 (207–486)	0.06	0.14
Rectus femoris	0.56 (0.39–1.64)	0.58 (0.41–0.77)	146 (90–459)	0.17	0.51
Soleus	2.40 (1.00–7.03)	2.34 (1.10–6.88)	1215 (536–4960)	0.80	0.88
Gastrocnemius medialis	1.50 (0.76–3.25)	2.31 (0.82–3.54)	393 (229–611)	0.14	0.14
Gastrocnemius lateralis	0.97 (0.72–3.04)	1.14 (0.63–4.23)	124 (55–377)	0.45	0.51
Tibialis posterior	1.42 (1.06–3.41)	1.18 (0.97–3.23)	140 (77–442)	0.07	0.29
Tibialis anterior	1.66 (1.02–4.40)	2.86 (1.07–5.07)	230 (117–879)	0.07	0.07

SUV: Standardized Uptake Value. AUV: Absolute Uptake Value. SUV and AUV values are given as median (range).

*Wilcoxon matched-pair signed-rank test (2 samples).

**Bold** face indicates a significant difference.

### Heterogeneity of FDG uptake

**Fig 2:**
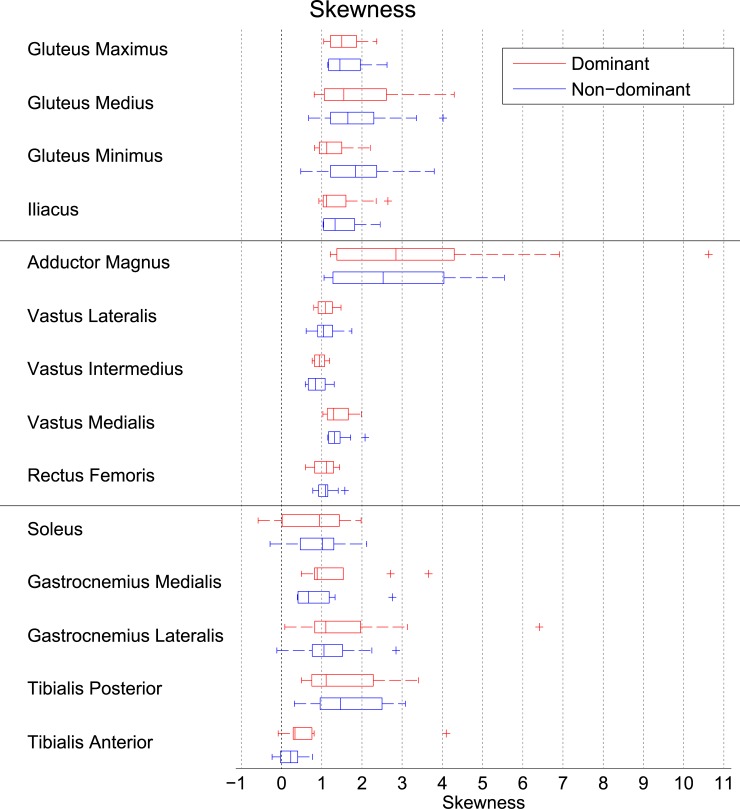
Skewness of the FDG uptake distribution in the muscles in the dominant and the non-dominant lower limb. A skewness of zero would indicate a normal, Gaussian distribution, whereas higher skewness values such as observed in the Fig indicate that the most frequently occurring FDG uptake values clustered at the lower end of the distribution and the tail pointed towards the higher scores. On each box, the central mark is the median, the edges of the box are the 25th (q1) and 75th (q3) percentiles, the whiskers extend to the most extreme data points not considered outliers, and outliers are plotted individually. Points are drawn as outliers if they are larger than q3 + 1.5*(q3 –q1) or smaller than q1–1.5*(q3 –q1).

**Fig 3:**
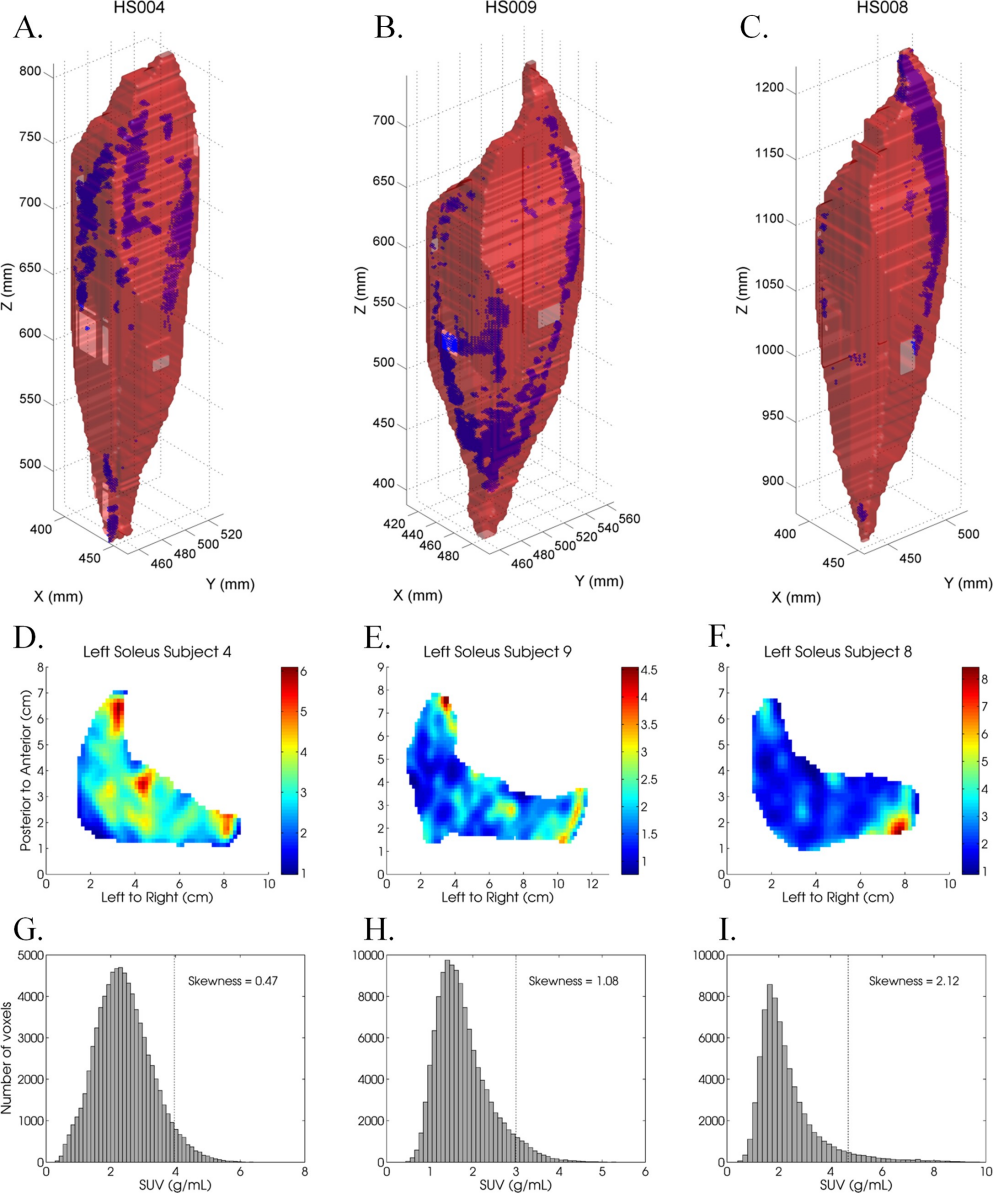
a-c: Three-dimensional rendering of the soleus muscle for three subjects, including marked ‘hot’ voxels in blue. The X, Y and Z-coordinates reflect the anterior-posterior, left-right and distal-proximal directions, respectively. They are shown in the DICOM patient coordinate system. d-f: Slice of the soleus muscle taken at 2/3 of its height, with colors indicating the uptake value (SUV, i.e. g/mL). The soleus in the middle was the largest; this subject was male, whereas subjects 4 and 8 were females. g-i: Histograms of the distribution of the entire muscle’s FDG uptake across the spectrum of uptake values. The histogram that corresponds with the lowest skewness value (on the left) is rather bell-shaped, whilst the other two clearly are not.

The SUVs of the individual voxels within muscles tended to be concentrated at the lower end of the histograms, with a tail towards the higher SUVs, as indicated by the median skewness values that were higher than zero in all muscles, and higher than one in 10 out of 15 muscles (Fig 2). Example histograms of muscles with low (0.5), typical (1.4) and high (2.1) skewness are shown in Fig 3G, 3H and 3I, respectively. The adductor magnus had the highest median skewness (2.8 and 2.5 for the dominant and non-dominant limbs, respectively), as well as the widest range of skewness values between subjects (ranging from 1.2 to 10.6 in the dominant limb). In addition, the gluteal muscles also exhibited relatively large skewness values. The tibialis anterior was the only muscle that was hardly skewed at all (median skewness 0.3 in the dominant limb and 0.2 in the non-dominant limb). From Fig 2 it is also evident that there were large inter-subject differences in the skewness values in the vast majority of muscles, with the exception of the quadriceps muscles and the tibialis anterior.

### Clustering of FDG uptake

**Fig 4:**
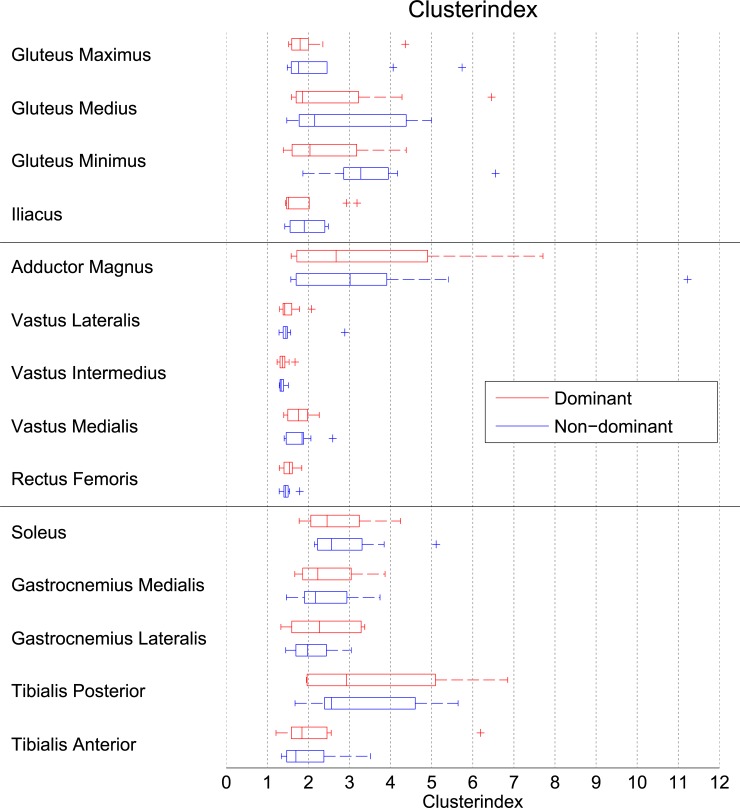
Cluster indices per muscle. A cluster index of zero would indicate a homogeneous distribution of the ‘hot’ voxels of FDG across the muscle, whereas higher values are indicative of a clustering effect. On each box, the central mark is the median, the edges of the box are the 25th (q1) and 75th (q3) percentiles, the whiskers extend to the most extreme data points not considered outliers, and outliers are plotted individually. Points are drawn as outliers if they are larger than q3 + 1.5*(q3 –q1) or smaller than q1–1.5*(q3 –q1).

In all muscles, voxels with high FDG uptake tended to cluster together rather than being distributed heterogeneously. This is indicated by the cluster index, which was higher than one for all muscles (Fig 4). The clustering was most pronounced in the adductor magnus, soleus, tibialis posterior and non-dominant gluteus minimus, with median cluster indices of two or more. Clustering was relatively low in the quadriceps muscles, as indicated by the median cluster indices of 1.3–1.8, and this observation was consistent across subjects. In contrast, the degree of clustering varied widely between subjects in most of the other muscles.

The locations of clustered areas also varied greatly between subjects for any given muscle. For example, the stretch of clustering that is visible in Fig 3B and 3C in the cranial, medial aspect of the soleus was present in four subjects, whereas the other six subjects had clustered areas elsewhere, or the clustered areas were smaller and more scattered throughout the muscle. Hence, we found clustering of high FDG uptake, but could not identify any consistent intra-muscular pattern of FDG uptake in any of the 15 muscles amongst the 10 healthy subjects included in this study.

## Discussion

This study aimed to determine asymmetries in [^18^F]-fluorodeoxyglucose uptake between muscles in the dominant and the non-dominant lower limb, as well as to determine heterogeneities in [^18^F]-fluorodeoxyglucose uptake within muscles during walking, using three-dimensional magnetic resonance imaging-based whole-muscle segmentations. Large inter-limb differences in uptake were found in the lower leg muscles and in the gluteus medius and gluteus minimus (differences up to 98% were found), whereas the uptake in the thighs was relatively symmetrical between the limbs. Thus, our first hypothesis was rejected. [^18^F]-fluorodeoxyglucose uptake was not systematically higher in the dominant or the non-dominant limb in any muscle, including when inter-limb muscle volume differences were taken into account. The skew of the standardized uptake values was to the right almost exclusively, indicating that most voxels had a relatively low standardized uptake value, and that a minority of voxels had a relatively high standardized uptake value. Thus, our second hypothesis was confirmed. Finally, rather than being homogeneously distributed in the muscle, the voxels with high standardized uptake value tended to cluster in virtually all muscles, the clustering being strongest in the adductor magnus, the ankle plantar flexors, and the gluteus medius and minimus. Thus, our third hypothesis was rejected.

Considering that the subjects were healthy, our finding that there were large asymmetries in muscle [^18^F]-fluorodeoxyglucose uptake between the limbs in several muscles was unexpected. According to traditional assumptions and a substantial body of literature (for overview, see [1]), gait in the absence of disease has been supposed and found to be symmetrical. However, this notion is still under debate [1], and the body of evidence supporting some asymmetry is also substantial. For example, in a large retrospective study (182 subjects), peak hip and knee flexion and adduction moments during walking exceeded 10% asymmetry in more than half of the participants [38]. Similar studies that looked at spatiotemporal parameters, kinematics, and ground reaction forces also found significant asymmetries in these parameters [2, 3, 32]. Asymmetries in such spatiotemporal, kinematic, and kinetic parameters are essentially the result of many muscles activating in intricate patterns to form the individual’s unique walking motion. It is, therefore, plausible that the individual contributors to this motion (i.e. the muscles) could also be activating in an asymmetric fashion. Besides the current study which used FDG-PET, there are also studies that found asymmetries in muscle activity using electromyography [4, 5]. Nevertheless, in the present study, the asymmetries in terms of glucose uptake of more than 25% and even 50% that we found in many muscles, particularly in the lower leg, were larger than in any literature.

A first possible explanation for the large asymmetries could be limb dominance, but the direction of the asymmetries in uptake varied widely between subjects and was not consistently pointing towards the dominant or the non-dominant limb in any muscle. A second explanation for a muscle being more active in one limb than in the contralateral limb, would be that it is compensating for similarly asymmetric co-activation in an antagonist muscle. Yet, we did not observe such a trend in the uptake data, and there were several subjects who actually showed an opposite effect. A third possible explanation for the large inter-limb asymmetries is that the muscles in either the dominant or the non-dominant limb would have different degrees of work efficiency. However, if this were the case, it would have been likely that a given muscle would consistently show a higher degree of efficiency (and thus, lower uptake) in either the dominant or in the non-dominant lower limb across subjects, which was not observed. Finally, a fourth explanation is that a very active muscle could be compensating for reduced activity in another muscle that has a similar functional role. For example, high activity in one of the plantar flexors (e.g. soleus) could be accompanied by a lower activity in the other plantar flexors (mainly medial and lateral gastrocnemius). Such a trend was not observed; subjects who had a high absolute uptake value in the soleus generally also had a high uptake in the medial and lateral gastrocnemius, and vice versa. Hence, it remains difficult to explain the large asymmetries, and this warrants further research using other methods (e.g. electromyography and kinematic and kinetic gait analysis) in conjunction with the [^18^F]-fluorodeoxyglucose-positron emission tomography technique.

Interestingly, the distribution of the standardized uptake values across the voxels comprising each muscle did not follow a Gaussian curve, but was instead skewed to the right in almost all muscles. This indicates that there was a relatively large number of voxels which exhibited a standardized uptake value that was substantially lower than the mean standardized uptake value of the entire muscle. This high degree of skew is in line with Heinonen et al., who used [^18^F]-fluorodeoxyglucose-positron emission tomography to examine the heterogeneity of glucose uptake in the quadriceps during exercise at different intensity levels [16]. Albeit in a different task (cycling), it was found that the heterogeneity of glucose uptake in the quadriceps was relatively high at low exercise intensity levels, and that it decreased with increasing exercise intensity levels (30%, 55% and 75% of maximum VO_2_). Since level walking at comfortable speed is generally considered a low-intensity exercise, the high degree of skew that we observed is in line with this literature [16]. The fact that skewness might be related to the intensity level of the activity opens the question whether it may be related to the fiber type composition of the muscle. This does not appear to be the case; the soleus has a very high type I fiber percentage of 87.7% [39] and exhibited an average degree of skew. Similarly, the rectus femoris, which has a very low type I fiber percentage of 38.1% (and, consequently, a high type II fiber percentage of 61.9%) [39], also exhibited only an average degree of skew. The adductor magnus and tibialis anterior, however, have lower percentages of type I fibers than the soleus (58.4% and 73.1%, respectively [39]), yet these exhibited the most (adductor magnus) and least (tibialis anterior) skewed distributions.

The voxels with high uptake of [^18^F]-fluorodeoxyglucose were clustered instead of being distributed homogeneously throughout the muscle, especially in the adductor magnus, the ankle plantar flexors, and the gluteus medius and minimus. This suggests that most of the work during walking is performed by limited regions within muscles. Since comfortable level walking is an aerobic activity, and since it is known that the different types of muscle fibers are organized in regions of different composition rather than being homogeneously distributed [40], the observed regional uptake differences could be due to regional differences in muscle fiber types. In conjunction, due to the size principle, the recruitment of motor units in theory causes region-specific muscle activation when a certain specific demand is placed on the muscle [14, 41]. In simple tasks (e.g. isometric exercises), this phenomenon of region-specific activation has been confirmed using modern techniques such as multi-channel electromyography [10, 41], magnetic resonance imaging [13] as well as [^18^F]-fluorodeoxyglucose-positron emission tomography [12, 15, 42]. Our results are in line with these findings and suggest that, also in a more complex task (walking), region-specific differences in muscle activation exist in almost all muscles.

Rather than forming consistent patterns across subjects, the spatial distribution of [^18^F]-fluorodeoxyglucose uptake in the muscles was very variable. Typically, three or four subjects could be grouped into one category of regional uptake (e.g. cranial, medial aspect in the soleus such as in Fig 3B and 3C), but no consistent patterns were observed across all subjects in any muscle. It is known that activation of anatomical regions within a muscle can be related to a specific functional task that needs to be performed [14, 43], or to mechanical demands [44] or force direction demands [10]. It also seems to be the case here that different spatial regions of muscles contribute differently to walking than others. Again, this warrants further research as to how and why these specific patterns differ between individuals and how they affect the walking pattern.

### Limitations

This study has some limitations. First, the inter-subject variation in standardized uptake values was high in some muscles, and the current sample size was too small to attribute this to any particular characteristic of these healthy subjects such as age, body mass index or general fitness level. However, high inter-subject variability in muscle activation during walking has been shown to exist with techniques such as electromyography as well [45–47]. Second, our experimental protocol did not include a control condition such as resting. Therefore, it was not possible to assess whether the observed intra-muscular heterogeneities in [^18^F]-fluorodeoxyglucose uptake were attributable to the walking task or (partly) due to varying levels of resting metabolism within muscles. Extending the protocol was not allowed by the medical ethical board at our hospital due to ethical constraints regarding the radiation dose to these healthy subjects. Furthermore, our measurement protocol did not include gait analysis. This would have allowed us to test our visual observation that the healthy subjects walked symmetrically. Third, our walking time was long (90 minutes), which has two disadvantages. One is in transitioning from research on healthy subjects to clinical applications, as patients may change their gait during the prolonged walking task due to pain or fatigue in certain muscles. The other disadvantage is that [^18^F]-fluorodeoxyglucose-positron emission tomography is a cumulative measurement that provides no temporal information. Therefore, activation of muscles and different regions within each muscle may have shifted during the task, which makes interpretation of the asymmetry and region-specific uptake results difficult. It is possible that the subjects activated their muscles more symmetrically at the onset of walking, and later shifted their strategy to (some) more asymmetrical muscle activations, or vice versa. Future work could focus on examining region-specific [^18^F]-fluorodeoxyglucose uptake in a simpler, more controlled task, or it could focus on combining [^18^F]-fluorodeoxyglucose-positron emission tomography with multi-channel electromyography, such that changes in motor unit recruitment and asymmetry can be tracked over time and coupled with the [^18^F]-fluorodeoxyglucose-positron emission tomography results.

## Conclusions

The volumetric analysis of [^18^F]-fluorodeoxyglucose uptake in both lower limbs after walking showed that muscle activity (in terms of glucose uptake) was symmetrical in the thigh muscles, whereas it was highly asymmetric in the lower leg and gluteal muscles in many subjects. These findings challenge the common assumption of symmetry between the limbs in healthy subjects. Practically all of the inter-subject differences disappeared when the results were averaged across subjects, which underlines that averaging individual subject data stemming from both limbs may preclude asymmetries from being noticed or reported. Studies that have the goal of validating muscle activation levels using subject-specific musculoskeletal models, will need to be cautious that muscle activity for the most active muscles during the activity (e.g. lower leg and gluteal muscles for walking) needs to be validated for each subject and each limb individually. As for the [^18^F]-fluorodeoxyglucose distribution within muscles, there was a majority of voxels that had a standardized uptake value that was lower than the mean standardized uptake value, and there was a minority of voxels with a relatively high standardized uptake value in almost all muscles. Those ‘hot’ voxels tended to cluster rather than being spread homogeneously throughout the muscle, but those clusters were not located in consistent locations in a given muscle across subjects. The inconsistent locations of ‘hot’ clusters highlights the added value of performing a three-dimensional analysis of uptake rather than examining uptake in only a single slice for each muscle. Future research could focus on revealing the relation of active versus resting [^18^F]-fluorodeoxyglucose uptake levels, on measuring kinematics and kinetics while the exercise is performed, and on combining the cumulative [^18^F]-fluorodeoxyglucose-positron emission tomography measurement with a temporal-sensitive method such as multi-channel electromyography. When applied in patients, FDG-PET offers the perspective of being able to show which muscles and which parts of muscles are activated less than ‘normal’, and which are more active and might be compensating for other muscles or for underlying pathology. This would be useful information that would be difficult to obtain through other methods. It could have benefit, for example, in studying patients who have undergone joint replacement, who have had an anterior cruciate ligament reconstruction, or who are suffering from neurological disease. Using [^18^F]-fluorodeoxyglucose-positron emission tomography to measure muscle activity remains an exciting new technique, which has the potential to further improve our understanding of muscle contributions during exercise in many ways, including in detecting differences in muscle activity levels between subjects, between limbs, and between regions within muscles.

## Abbreviations

FDG: [^18^F]-fluorodeoxyglucose
PET: positron emission tomography
CT: computed tomography
MRI: magnetic resonance imaging
SUV: standardized uptake value
ROI: region of interest
ITK: Image Segmentation and Registration Toolkit
DICOM: Digital Imaging and Communications in Medicine
ASI: absolute symmetry index
SI: symmetry index
SUV: standardized uptake value
AUV: absolute uptake value
NHN: number of hot neighbors
